# Topical application of nebulized human IgG, IgA and IgAM in the lungs of rats and non-human primates

**DOI:** 10.1186/s12931-019-1057-3

**Published:** 2019-05-22

**Authors:** Cédric Vonarburg, Marius Loetscher, Martin O. Spycher, Alain Kropf, Marlies Illi, Sharon Salmon, Sean Roberts, Karin Steinfuehrer, Ian Campbell, Sandra Koernig, Joseph Bain, Monika Edler, Ulrich Baumann, Sylvia Miescher, Dennis W. Metzger, Alexander Schaub, Fabian Käsermann, Adrian W. Zuercher

**Affiliations:** 1 CSL Behring AG, Research, Bern, Switzerland; 2 Department of Immunology and Microbial Disease, Albany Medical College, Albany, NY 12208 USA; 3 PARI Pharma GmbH, Lochhamer Schlag 21, 82166 Gräfelfing, Germany; 4 CSL Ltd., Research, Bio21 Institute, Parkville, Victoria Australia; 5 CSL Behring GmbH, Pharmacology and Toxicology, Marburg, Germany; 6 Medizinische Hochschule Hannover, Hannover, Germany

**Keywords:** Inhalation, Plasma-derived immunoglobulins, Polymeric immunoglobulins, Topical application, Non-human primates

## Abstract

**Background:**

Recurrent and persistent infections are known to affect airways of patients with Primary Immunodeficiency despite appropriate replacement immunoglobulin serum levels. Interestingly, patients with Chronic Obstructive Pulmonary Disease or with non-CF bronchiectasis also show similar susceptibility to such infections. This may be due to the limited availability of immunoglobulins from the systemic circulation in the conductive airways, resulting in local immunodeficiency. Topical application of nebulized plasma-derived immunoglobulins may represent a means to address this deficiency. In this study, we assessed the feasibility of nebulizing plasma-derived immunoglobulins and delivering them into the airways of rats and non-human primates.

**Methods:**

Distinct human plasma-derived immunoglobulin isotype preparations were nebulized with an investigational eFlow® nebulizer and analyzed in vitro or deposited into animals. Biochemical and immunohistological analysis of nebulized immunoglobulins were then performed. Lastly, efficacy of topically applied human plasma-derived immunoglobulins was assessed in an acute *Streptococcus pneumoniae* respiratory infection in mice.

**Results:**

Characteristics of the resulting aerosols were comparable between preparations, even when using solutions with elevated viscosity. Neither the structural integrity nor the biological function of nebulized immunoglobulins were compromised by the nebulization process. In animal studies, immunoglobulins levels were assessed in plasma, broncho-alveolar lavages (BAL) and on lung sections of rats and non-human primates in samples collected up to 72 h following application. Nebulized immunoglobulins were detectable over 48 h in the BAL samples and up to 72 h on lung sections. Immunoglobulins recovered from BAL fluid up to 24 h after inhalation remained structurally and functionally intact. Importantly, topical application of human plasma-derived immunoglobulin G into the airways of mice offered significant protection against acute pneumococcal pneumonia.

**Conclusion:**

Taken together our data demonstrate the feasibility of topically applying plasma-derived immunoglobulins into the lungs using a nebulized liquid formulation. Moreover, topically administered human plasma-derived immunoglobulins prevented acute respiratory infection.

**Electronic supplementary material:**

The online version of this article (10.1186/s12931-019-1057-3) contains supplementary material, which is available to authorized users.

## Background

Primary immunodeficiency diseases (PID) comprise many rare and heterogeneous disorders in which part of the body’s immune system is missing or not functional [1, 2]. In many PID patients, plasma immunoglobulins (Igs) are present at low levels or absent, leading to a high susceptibility to severe bacterial infections such as pneumonia. To overcome reduced antibody production, PID patients receive plasma-derived IgG as first line therapy [3, 4].

While pneumonia events have decreased under optimized IgG substitution therapy [5], recurrent and persistent infections in the airways are still widely observed in PID patients and can eventually contribute to the development of chronic lung disease, one of the main causes of mortality in this population [5–8].

While plasma proteins, including IgG, can reach the alveoli through transudation [9], movement of plasma-derived Igs into the conducting or upper airways has not been described. Surveillance of these regions is mainly conducted by local components of the mucosal immune system. Thus, assuming that PID patients under IgG substitution therapy would still suffer local immunodeficiency, we hypothesized that protection of these areas of the respiratory tract might be best achieved by direct application of Igs via inhalation.

Chronic obstructive pulmonary disease (COPD) patients have normal systemic immunity. However, as a consequence of the disease, tissue remodelling of the bronchi has been associated to a reduction in secretory IgA (sIgA) levels at the mucosal surfaces of these patients [10, 11]. In addition, sIgA from submucosal glands are snared into mucus plugs [12]. These processes may then result in local immunodeficiency of the airways of COPD patients.

Several approaches are used to deliver proteins into the lungs. Nebulizers are the most commonly used inhalers as they allow the delivery of a higher dose of drug into the lungs [13–15]. However, there is limited information on the aerosolizing of Igs with nebulizers [16–18].

Building on recent improvements in both device technology and drug formulation, we have assessed the feasibility of nebulizing highly concentrated plasma-derived Igs (50 mg/ml to 100 mg/ml) using a state-of-the-art active membrane nebulizer. Following the demonstration that the nebulization process left the antibody molecules intact and active, we topically applied these aerosols into the lungs of rats and non-human primates and recovered intact and functional Igs 24 h and 48 h, respectively, from lung washes of treated animals. In addition to IgG, we also compared IgA and IgM preparations as these Ig isotypes are also important effectors of pathogen neutralization at mucosal sites. At last, we assessed the efficacy of human plasma-derived immunoglobulins for preventing acute respiratory infection in mice.

## Methods

### Preparation of human plasma-derived Igs

Human plasma-derived Igs were prepared as previously reported [19, 20]. IgA and IgAM are obtained from an ion-exchange chromatography step used in the large-scale manufacture of IgG from human plasma. IgAM contains IgA and IgM in a 2:1 mass ratio. Elution fractions containing IgA (IgA and IgAM, respectively) were concentrated and to 50 mg/ml protein in PBS by tangential-flow filtration (TFF; Pellicon XL Biomax 30, Merck Millipore) and subsequently re-formulated at 50 mg/ml protein in 125 mM proline. Of note, IgG products are composed of 97% monomeric IgG and concentrated at 100 mg/ml (IgPro10, Privigen®). For some experiments IgPro10 was diluted with water to obtain a 50 mg/ml final concentration. IgA preparation is 98% IgA. IgAM contains at least 98% of Ig. 50% of IgA in IgAM preparation are of dimeric form. IgM is of pentameric form.

### Aerosol generation and characterization

Nebulization of Ig preparations was performed with an investigational electronic vibrating membrane nebulizer (eFlow®; PARI Pharma GmbH, Germany). The nebulizer used for the study is based on the eFlow® technology and includes an optimized membrane designed to generate a high output rate as well as a big mixing chamber (not yet commercially available). Droplet sizes (Mass Median Diameter (MMD)) and the droplet size distribution (Geometric Standard Deviation (GSD) measurements were conducted at PARI Pharma GmbH (Graefelfing, Germany) using laser diffraction (Malvern MasterSizerX). Test conditions were as followed: airflow: 20 L/min ± 1.0 L/min; temperature: 23 °C ± 2 °C; relative humidity: 50% ± 5%.

### SEC and SDS-page

Aerosol droplets were collected in polypropylene tubes. Structural integrity and multimerization of nebulized Ig preparations were analyzed using SDS-PAGE and size exclusion chromatography (SEC). SDS-PAGE was performed using the Mini-Cell system of Life Technologies, according to the manufacturer’s protocols. Samples were denatured in sample buffer under reducing or non-reducing conditions, respectively. Electrophoretic separation was carried out on pre-cast gradient gels, NuPAGE Novex™ Bis-Tris 4–12% 1.0 mm 15 well, using NuPAGE™ MES electrophoresis buffer (Life Technologies). After electrophoresis, proteins in the gels were fixed and stained with Coomassie G-250 (SimplyBlue Safestain™; Life Technologies) according to the manufacturer’s protocol. The protein staining pattern was digitally recorded using an ImageQuant™ LAS 4000 system (GE Healthcare Lifesciences).

For SEC analysis, 2 μL of undiluted samples were injected into an Agilent Technologies 1260 Infinity™ HPLC system for size exclusion chromatography at a flow rate of 0.7 ml/min over a TSK gel G3000SWxl 7.8 mm ID × 30 cm column (Tosoh Bioscience). From the resulting chromatograms the relative contents of Igs polymers/high molecular weight structures, dimers, monomers and fragments were assessed.

### Ig functions

Fc activity: Respiratory burst was measured in purified human neutrophils. Rabbit erythrocytes (5 × 10^6^/ml) were incubated for 1 h at room temperature with increasing amounts of the different Ig preparations (0–800 μg/ml). After washing they were added to purified human neutrophils (~ 5 × 10^5^/ml). Respiratory burst was measured by chemiluminescence after adding a luminol solution (Luminol Na-Salt, Sigma) to the cells and recording at 37 °C over 90 min. The area under the signal-to-time curve was calculated and translated as RLU (relative light units). In parallel, the amount of Ig binding the erythrocytes was determined by flow cytometry (anti-human IgG (Nordic), FACS Canto II, Becton Dickinson). Binding and chemiluminescence data were then represented on the Y and X axis of a graph, respectively. For every sample, the amount of Ig bound to the erythrocytes to induce half-maximum respiratory burst of the (pre-nebulisation) control sample was determined. Fc activity was calculated as a ratio of the control sample according to the formula: Fc activity (sample) = half maximum chemiluminescence control/half maximum chemiluminescence sample.

### Antigen binding assay

Nebulized and non-nebulized Igs were assayed against Influenza A Haemaglutinin (H1) (Abcam), Influenza A Neuraminidase (N1) (Abcam), Pneumococcal Polysaccharide Type 1(PnP1) (ATCC; 161-X), Pneumococcal Polysaccharide Type 3(PnP3) (ATCC; 32-X) and Tetanus Toxoid Antigen (TTA). H1 (1μg/ ml; 3 h at 37 °C), N1 (1μg/ ml; 3 h at 37 °C), PnP1 (5 μg/ ml; 1 h at 37 °C), PnP3 (5 μg/ ml; 1 h at 37 °C) and TTA (5 μg/ml; 1 h at 37 °C) were coated into wells of a 96 well plate for the indicated times. After wash with PBS-Triton X100 (0.05%), wells were blocked as followed. For H1 and N1, Smart Block (Candor) was used for 2 h at 37 °C. For PnP1 and PnP2, 2,5% Fetal Bovine Serum (FBS) (GIBCO) was used for 1.5 h at RT. At last, for TTA, Superblock (Thermo scientific) was used for 1 h at 37 °C. Following additional washing steps with PBS-Triton, diluted Ig samples (50 μg/ml in PBS) were added to the wells and incubated at RT for 1 h and further washed three times with PBS-Triton.

For detection, HRP-conjugated goat anti-human IgG/A/M F(ab)‘2 HRP (1:2000 in PBS, Novex) was added for 2 h. Following five consecutive washes with PBS-Triton, chromogenic substrate (TMB, Fitzgerald) was added for 3-10 min and reaction was stopped with 50 μL HCl 1 M (Merck). Absorbance was read at 450 nm with reduction at 630 nm using an ELISA Plate Reader (Perkin Elmer, Envision Xcite).

Broncho-alveolar lavage (BAL) samples from the rat and NHP studies were selected for their equal content in IgG (50 μg/ml) to facilitate their comparison in an ELISA. Samples from vehicle-treated animals were used as negative control and as positive control, when spiked with 50 μg/ml non-nebulized IgG. Samples were measured in triplicates in TTA and PnP ELISAs as described above.

### *Streptococcus pneumoniae* A66.1 type 3 (Spn3) binding assay

To determine the total anti-Spn3 titer in the various preps, a PolySorp plate (Thermo Fisher Scientific #475094) was coated with 5x10^7^CFU/ml *Strep pneumoniae* A66.1 and incubated overnight at 4 °C. After washing twice with 0.05%Tween/PBS, the plate was blocked with 150 μl of 2.5% FBS/PBS. Samples diluted 2-fold were added after washing twice. Following 1.5 h incubation, the plate was washed and bound antibody was detected using goat anti-human IgG/A/M(total)-HRP diluted 1/16 k. The plate was developed using TMB substrate (BD Biosciences #555214), stopped with 1.8 N sulfuric acid, and read at 450 nm.

### Breath simulation experiments

Breathing simulation experiments were conducted at PARI Pharma GmbH. Adult breathing pattern was used according to Ph. Eur. 2.9.44 (i.e. a tidal volume of 500 ml, 15 breaths per minute and an inhalation: exhalation (I:E) ratio of 50:50). An inspiratory filter (polypropylene; PARI Pharma GmbH) was inserted onto the mouth piece, between the nebulizer and the pump.

To determine the delivered dose, 2 ml of Ig preparation were nebulized until aerosol was no longer visible. Aerosol droplets were collected on the inspiratory “inhalation” filter. The filter was rinsed with buffer containing 0.9% saline and 0.5% SDS (sodium dodecyl sulphate, 98.5%) in purified water. The filter was extracted for 1 h while shaking on a rotator. Using UV spectrophotometry, and according to the Beer-Lambert law (A = ε·c·L) using the mass absorption coefficient of ε(0.1%) = 1.38 ml/(mg∙cm), amount of Igs on the filter (delivered dose) could be calculated.

The respirable doses were calculated on the basis of the delivered dose and the mean respirable fractions determined by laser diffraction.

### Pharmacokinetic studies

Pharmacokinetic studies were performed at Charles River Laboratories (Edinburgh, UK).

Topical application of vehicle, nebulized IgG, IgA and IgAM (50 mg/ml, formulated in 125 mM proline) was assessed in both rats (Sprague-Dawley rat) and non-human primates (NHP; Cynomolgus Monkey). Thirty rats per group (120 total; *N* = 30/group) were exposed to a single snout-only inhalation exposure (15 min) while 4 NHP per group were exposed to facial (mask) inhalation exposure once weekly (30 min), for 4 weeks in a row.

Achieved doses were estimated as published using the following criteria [21]: Total Delivered Dose (mg/kg/day) = (C x RMV x T)/BW, Delivered Lung Dose (mg/kg/day) = (C x RMV x T)/BW x DF, Lung Dose (mg/g lung tissue) = (C x RMV x T)/LW x DF, where C = Aerosol concentration (mg/L of test item), RMV = Respiratory minute volume (L/min) (rat: 0.187 L/min; NHP: 1.550 L/min), T = Duration of dosing (min), BW = Animal body weight (kg), DF = Lung deposition factor (taken as 10% of inhaled dose), and LW = Lung weight.

The mass aerosol concentration of test aerosol in the animals’ breathing zone was measured gravimetrically and test aerosols were sampled through appropriate filters. The filters were weighed before and after sampling and the aerosol concentration calculated using the weight of test item collected and the volume of air sampled (aerosol flow rate of 0.5 L/min).

Sample collection was set up as follows. In the rat study, 6 animals were euthanized at each time point post-exposure (immediate post-dose (IPD), 1 h, 6 h, 12 h, and 24 h). For each time point, plasma collection and broncho-alveolar lavage were performed for 3 rats per group while the 3 remaining rats had their lungs collected for immunohistology. For BAL collection, trachea was cannulated and the lung lavaged two times with sterile PBS (2 × 1 ml). BAL samples were centrifuged (at 1500 *x* g for 10 min at approximately 4 °C) and the BAL supernatants were aliquoted and stored frozen (approximately − 80 °C) until analysis.

For the NHP study, a total of 16 animals was used and 4 groups studied: vehicle, IgG, IgA, IgAM. Each group was composed of four animals. Four time points post-exposure were analysed: immediate post-dose (IPD), 6 h, 24 h and 48 h. One animal/group was assessed at each time point. Sample collection included plasma and BAL. Anaesthesia of the animals was used during BAL collection. Three separate aliquots of sterile PBS (3 ml/kg per wash) were instilled through a syringe connected to the feeding tube sampling tube (catheter). Withdrawal of the fluid was performed on the same catheter. BAL were then centrifuged (at 1500 *x* g for 10 min at approximately 4 °C) and supernatants were aliquoted and stored frozen (approximately − 80 °C) until analysis.

Urea nitrogen concentrations were determined in both plasma and BAL at Charles River Laboratories using a commercial microplate-based colorimetric assay (BQ kits, USA). The ratio of these two concentrations gives the BAL dilution factor. This factor can be applied on BAL Ig concentrations to obtain an estimation of the Ig concentration in the epithelial lining fluid before lavage.

### Ig levels in plasma and broncho-alveolar lavages

Detection of human Igs in rat samples: all samples were diluted at 1/100. Coating, blocking and detection steps were of 1 h each (at room temperature). Sample incubation was of 2 h at room temperature. For IgG detection, goat anti-human IgG antibody (SP1032; Acris) was coated onto a microplate (NUNC, Maxisorp) at 1.5 μg/ml final concentration in PBS supplemented with 1.6% BSA (which also served as blocking buffer). Goat anti-human IgG detection antibody (SP1032HRP; Acris) conjugated with Horse Radish Peroxidase was used at a 0.3 μg/ml final concentration (low cross buffer (Candor), 1% casein buffer). For IgA detection, goat anti-human IgA antibody (A80-202A; Bethyl) was coated onto a microplate (NUC, Maxisorp) at 0.5 μg/ml final concentration in PBS supplemented with 1% BSA (which also served as blocking buffer). Goat anti-human IgA detection antibody (A80-202P; Bethyl) conjugated with horse radish peroxidase was used at a 0.3 μg/ml final concentration (low cross buffer (Candor), 0.1% casein buffer). For both ELISA, tetramethylbenzidine was added to the wells for 10 min at room temperature. After stopping the reaction, signal was measured at OD = 450 nm. Lower limit of quantitation (LLOQ) value was of 0.5 μg/ml.

### Detection of human Igs in NHP BAL and serum samples by LC-MS

NHP BAL sample (21 μL) was placed into a clean Eppendorf tube followed by the addition of 50 mM NH_4_HCO_3_ / 9 mM DTT containing heavy-isoptope labeled peptides, which are specific for human Igs and used as internal standards. After incubation at 95 °C under 550 rpm on a heatblock shaker for 5 min, samples were alkylated by adding 0.1 M IAA and incubated for 20 min at RT protected from light. Tryptic digestion was carried out at 37 °C/550 rpm for 4 h and the samples were separated immediately on a C18 column (AdvanceBio Peptide Mapping, 2.1 × 150 mm). The measurements were conducted using an Agilent 6550 iFunnel QTOF mass spectrometer connected to an Agilent 1290 Infinity II HPLC instrument.

Data was analyzed by calculating the peak area of the analyte and the internal standard using Agilent MassHunter Quant software. A standard curve was created by Agilent MassHunter Quant where the ratio of the analyte response to the internal standard response was plotted against the concentration. LLOQ value was of 2.36 μg/ml for IgG1, 2.11 μg/ml for IgA1 and 4.26 μg/ml for IgM.

For serum analysis, LLOQ value for IgG1 was 5.64 μg/ml and for IgA1, 16.09 μg/ml.

### Immunohistology

Rats and NHP were sacrificed and lungs were isolated, fixed and embedded in paraffin for histology analysis. Distribution of IgG (for rat and NHP samples) and IgA (for NHP samples) in the lungs was then assessed using immunohistochemistry methods with specific secondary antibodies on the paraffin sections. Immunohistochemistry (IHC) evaluation was performed by a board-certified veterinary pathologist at Charles River Laboratories (Edinburg, UK).

### Murine strains

C57BL/6 wild-type were obtained from the Jackson Laboratory. Breeding was performed at Albany Medical College following Institutional Animal Care and Use Committee guidelines. Human CD89Tg mice (C57BL/6 background) were generated by Dr. Pawel Pelczar (University of Zurich, Switzerland) under contract. In a first strain, the *CD89* gene (Gene Reference: X54150.1) under CMV promoter regulation was inserted in oocytes (CD89^tg/wt^). A loxP-flanked mCherry sequence including a STOP codon between the CMV promoter sequence and the CD89 sequence prevented CD89 expression. Myeloid specific CD89-expressing mice were generated by crossing CD89^tg/wt^ and C57BL/6 LyzM^cre/cre^ Tg mice (licenced from Jackson Laboratories). Excision of the loxP-flanked mCherry cassette in vivo lead to CD89 expression in the myeloid cell lineage under a CMV promoter in 50% of the offspring (CD89^tg/wt^ / LyzM^cre/wt^). Breeding was performed at the University of Melbourne Bio21 Institute animal facility under specific pathogen-free conditions. Mice were sent to Albany Medical College for infection studies.

### *Streptococcus pneumoniae* (*S. pneumoniae*) respiratory infection

C57BL/6 wild-type and human CD89Tg mice were used for this infection model. All mice were treated intranasally under anesthesia with vehicle or Ig preparations (50 μl/mouse (e.g.: 1.33 mg of Ig/mouse)) at Day − 1. To induce pneumonia, mice were anesthetized on Day 0 and were treated intranasally with 5 × 10^5^ CFU of A66.1 *S. pneumoniae* strain. Anesthesia was obtained using ketamine HCl (Fort Dodge Animal Health, Fort Dodge, IA) and xylazine (Phoenix Scientific, St. Joseph, MO). Survival was monitored for 22 days.

### Statistical analysis

Graphpad Prism 7.0 (GraphPad Software, San Diego, CA, USA) was used for statistical analyses and graphs. All data are expressed as mean ± standard error of the mean. Statistical comparisons were performed using 1-way Anova test. Survival analyses used the Mantel-Cox log rank test. Statistical significance was defined as a *P* value < 0.05.

## Results

### Highly concentrated Ig preparations can be nebulized

We tested the feasibility of nebulizing different plasma-derived Ig preparations. Four preparations were tested. IgG, IgA and a mix of IgA and IgM (IgAM). Aerosol generation was observed for each formulation (Table 1) and nebulization of 2 ml samples occurred in less than 3.5 min. The total output rate of the device was inversely correlated to the viscosity of each Ig preparation, with the 5%-formulated samples giving the best output rate and the shortest nebulization time (Table 1).

**Table 1:** Aerosol characterization upon nebulization of distinct Ig formulations. GSD: Geometric Standard Deviation (Width of deviation curve of droplet sizes)

Formulation	Concentration (mg/ml)	Viscosity (20 °C) (mPa*s)	Total Output Rate (mg/min)	Mass Median Diameter (mm)	GSD	Respirable Fraction < 5 μm (%)	Nebulization time (min) (2 ml)
Saline 0.9%	–	~ 1.09	1072 ± 225	4.35 ± 0.25	1.76 ± 0.04	59.89 ± 4.29	n.d. (~ 1.8–2.0 min)
IgG 10%	100	3.2 ± 0.03	602 ± 88	3.74 ± 0.07	1.53 ± 0.07	76.87 ± 2.04	3 ± 0.4
IgG 5%	50	1.77 ± 0.01	812 ±132	3.98 ± 0.09	1.60 ± 0.02	69.68 ± 2.33	2.2 ± 0.4
IgA 5%	50	2.31 ± 0.01	700 ± 90	3.86 ± 0.10	1.57 ± 0.03	73.13 ± 2.97	2.6 ± 0.3
IgAM 5%	50	3.74 ± 0.02	681 ± 38	3.73 ±0.11	1.55 ± 0.03	76.46 ± 3.24	2.4 ± 0.2

Optimal deposition of an aerosol into the lungs requires the droplets to have a diameter below 5 μm [14]. Using laser diffraction, we found that the mass median diameter (MMD) was similar for all Ig preparations with a size below 4 μm (Table 1). The respirable fraction (RF), defined by the frequency of droplets with a diameter size below 5 μm, was above 69% for all Ig formulations (Table 1). Similar data were obtained when Ig products were formulated in PBS (see Additional file 1: Figure S1). Taken together these data demonstrate that it is feasible to efficiently aerosolize Igs, including very large molecules such as IgM (970 kD).

### Structural characterization of nebulized Igs

**Fig. 1:**
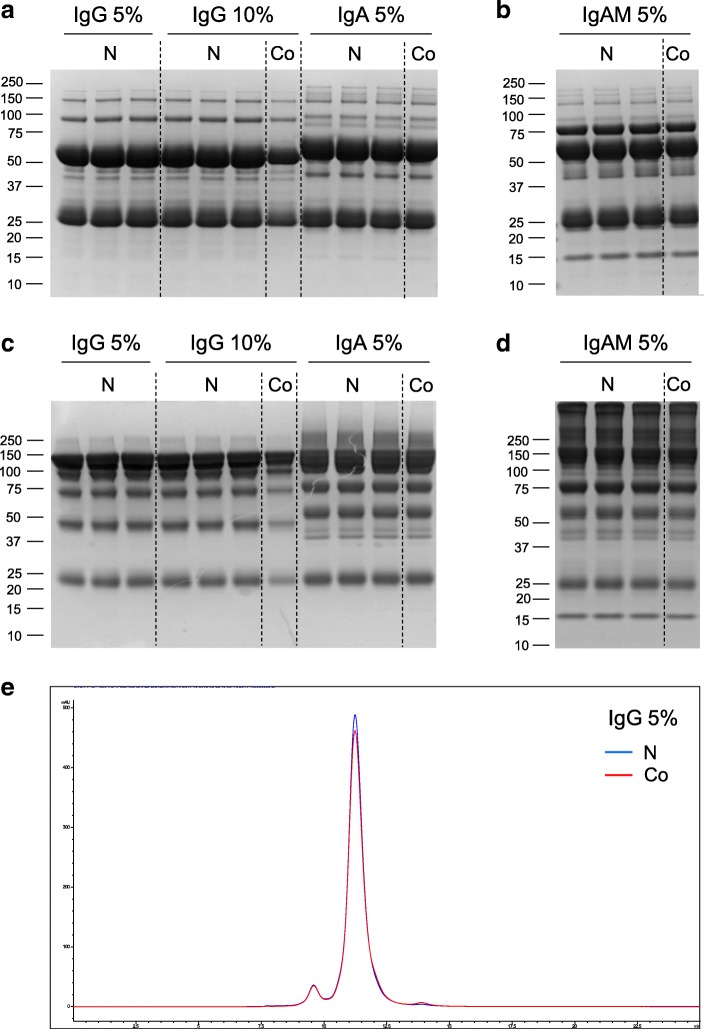
Assessment of Ig stability post-nebulization. **a-d** SDS analysis of control (Co) and nebulized (N) Igs were performed under reduced (**a**) and non-reduced (**c**) conditions. Similar analyses were done for polymeric Ig-enriched formulations under reduced (**b**) and non-reduced (**d**) conditions. **e** Chromatogram of IgG (5%) separated by size exclusion. Blue chromatogram refers to the nebulized (N) formulation. Red chromatogram refers to the non-nebulized, or control (Co), formulation

Nebulization of liquid biopharmaceuticals has been reported to induce physical stress, such as shearing force, drying and increased temperature, potentially leading to denaturation or aggregation [22]. Aerosol droplets were collected and analyzed by SDS-PAGE under reducing and non-reducing conditions in order to assess the impact of nebulization on the structure and the function of the Igs. The band patterns obtained from non-nebulized and nebulized Ig formulations on the gels were virtually identical for all preparations (Fig. 1, a-d), suggesting that Igs remained intact post-nebulization. This result was confirmed by size exclusion high-performance liquid chromatography (SE-HPLC) which yielded practically identical chromatograms pre- and post-nebulization (Fig. 1, e; Additional file 1: Figure S2A-B; Table 2). Similarly, Ig preparations with an increased content of high-molecular weight protein species, such as IgA (~ 25% Ig polymers (dimeric IgA) & aggregates) and the IgAM (~ 62% Ig polymers (dimeric IgA and IgM) & aggregates) preparations were not significantly altered by the nebulization process (Table 2). Further analysis of the IgAM preparations by dynamic light scattering (DLS) confirmed that nebulization did not alter particle size distribution (data not shown). In summary, nebulization of highly concentrated Ig preparations with an active vibrating membrane nebulizer is considered to a very limited impact on Ig structure.

**Table 2:** Assessment of Ig stability upon nebulization by size exclusion chromatography. Ig formulations were nebulized or left untreated and then analyzed by size exclusion chromatography. Percentage of fragments, monomers, dimers, polymers and high molecular species (HMS) are reported

Formulation	Condition	Polymers & HMS (%)	Monomers and Dimers (%)	Fragments (%)
IgG 10%	non-nebulized	< 1	> 98	< 1
	nebulized	< 1	> 98	< 1
IgG 5%	non-nebulized	< 1	> 98	1
	nebulized	< 1	> 98	< 1
IgA 5%	non-nebulized	23	74	3
	nebulized	23	74	3
IgAM 5%	non-nebulized	62	34	4
	nebulized	60	35	5

### Functional characterization of nebulized Igs

**Fig. 2:**
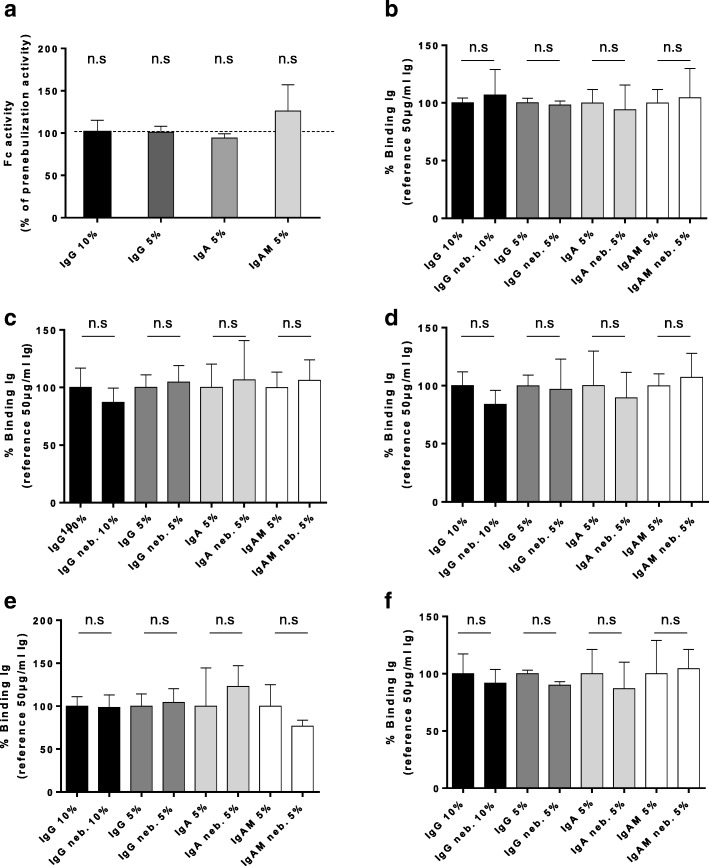
Evaluation of Fc and Fab activities of nebulized Igs. **a** Respiratory burst of neutrophils challenged with rabbit erythrocytes coated with nebulized or non-nebulized Igs was tested. Results are represented as the percentage of Fc activity of non-nebulized Igs. **b-f** Tetanus toxoid antigen (**b**), pneumococci polysaccharides serotype 2 (**c**) and 3 (**d**), influenza H1N1 hemagglutinin (**e**) and neuraminidase (**f**) were coated into wells. Binding of nebulized and non-nebulized Igs was assessed by ELISA. Results of non-nebulized Igs are represented as 100% activity

To address Fc function we used an assay based on Fc receptor-dependent activation of polymorphonuclear neutrophils with defined complexes of the Ig preparations. No significant differences were found between nebulized and non-nebulized immunoglobulin formulations in this assay (Fig. 2a).

Fab-based binding capacity of nebulized Igs was assessed by enzyme-linked immunosorbent assays (ELISA) for pneumococcal polysaccharides, tetanus-toxoid antigen, influenza A neuraminidase (N1) and hemagglutinin (H1) antigens. No significant differences of binding to the antigens were noted between the nebulized and non-nebulized Ig formulations (Fig. 2b-f). Polymeric immunoglobulins have been shown to be efficacious at preventing infection of Caco-2 cells with *Shigella flexneri* in vitro [19]. In this model, nebulized polymeric Igs showed the same potency as non-nebulized polymeric Igs in protecting the cell monolayer against infection and in maintaining Caco-2 responsiveness with a low pro-inflammatory profile (see Additional file 1: Figure S3). In summary, the biological functions of plasma-derived Igs were considered not to have been altered by the nebulization process.

### Application of nebulized Igs in vivo

**Fig. 3:**
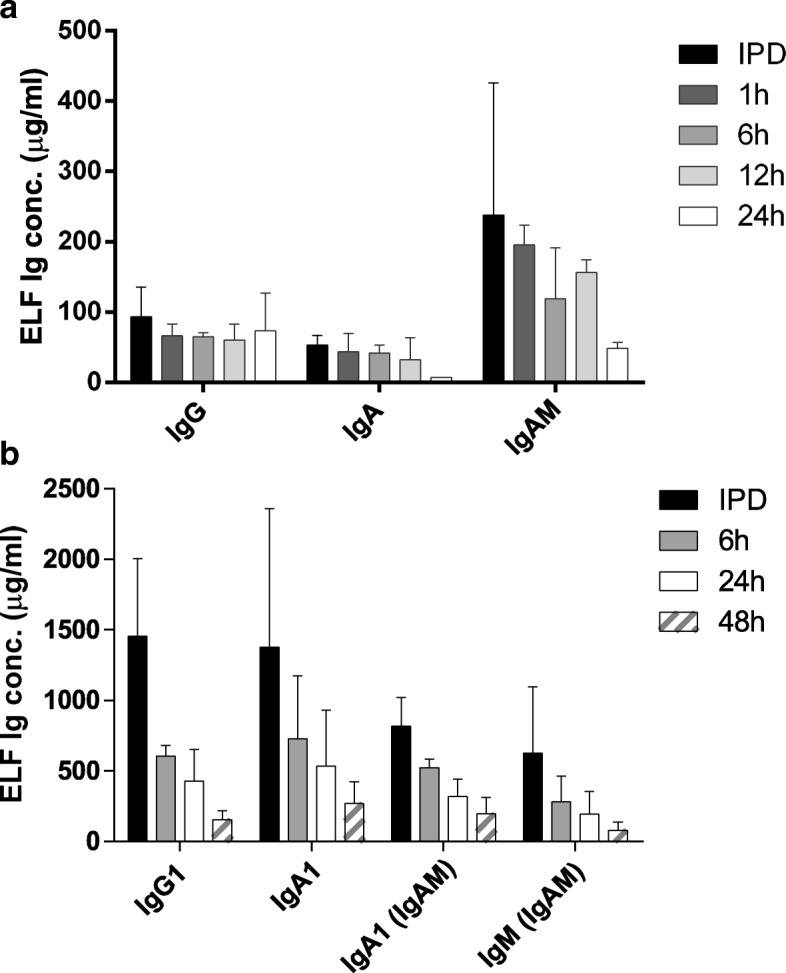
Ig levels in epithelial lining fluid of treated animals. **a-b** Ig levels were measured in both broncho-alveolar lavages and in plasma from rats (**a**) and non-human primates (**b**) collected at different time points post-inhalation. As depicted, kinetics ranged up to 24 h in rats and 48 h for non-human primates post-inhalation. Epithelial lining fluid concentration for each broncho-alveolar sample was calculated by applying a correction factor, obtained by dividing nitrogen urea plasma concentration by its concentration in broncho-alveolar lavage. Plasma Ig levels could not be quantified. Data are below the LLQ (5.64 μg/ml for IgG1 and 16.09 μg/ml for IgA1). There are therefore not presented. IPD: immediate post-dose

Our next goal was to test lung pharmacokinetics (PK) and biological distribution of aerosolized Ig in rats and non-human primates (NHP) (Table 3 and Fig. 3). Analysis of the aerosol concentration on filters placed in the aerosol chambers at a position comparable to the animals’ breathing zone gave an estimation of the overall delivered dose level in each species. In rats, it ranged from 31.7 to 38.5 mg/kg/day, in NHP from 25.6 to 29.6 mg/kg/day (Table 3). Immediately post-inhalation, broncho-alveolar lavages (BAL) were performed and Ig concentrations were measured in BAL fluid using either ELISA (rat) or liquid chromatography-mass spectrometry (LC-MS) (NHP) methods (Table 3 and Fig. 3); data were normalized using a factor based on the ratio of urea nitrogen BAL/plasma concentrations, which allows to estimate the epithelium lining fluid dilution by the introduced liquid during the BAL procedure [23]. The highest amount of Igs post-inhalation was between 2.4 (IgA) and 10.7 μg (IgA in IgAM) for the rats and 2454.6 (IgA1), 4368.4 (IgG1) and 1723.9 μg (IgM in IgAM) for the NHP (Table 3). The Ig-delivered dose in NHP was much higher than in rats, although the calculated delivered dose was higher in rats. No accumulation of nebulized Igs was observed over 4 inhalation cycles with a 1-week wash-out period between each cycle (data not shown).

**Table 3:** Pharmacokinetic study design, characteristics and immediate post dose (IPD) BAL Ig recovery. For the NHP study, isotype measured by LC-MS is indicated into brackets

Species	Product	Overall Estimated Delivered Dose Level (mg/kg/day)	Highest IPD BAL recovery (μg)
Rat	Vehicle	N/A	N/A
	IgG 5%	31.7	4.2
	IgA 5%	32.66	2.4
	IgAM 5%	38.54	10.7
NHP	Vehicle	N/A	N/A
	IgG 5%	29.6	4368.4 (IgG1)
	IgA 5%	25.8	2454.6 (IgA1)
	IgAM 5%	25.6	4130.1 (IgA1)
			1723.9 (IgM)

### Pharmacokinetics of nebulized Igs

BAL and plasma samples were collected at different time points post-inhalation. Pharmacokinetics were studied over 24 h in rats and over 48 h and in NHP. Detection of human Igs in BAL and plasma samples was conducted as described above, using either ELISA or LC-MS methods. Analysis of the PK of Igs in rat BAL showed that IgG and IgA were detectable for at least 24 h (Fig. 3a-b). In NHP, the main allotypes, IgG1 and IgA1, as well as IgM were detectable for up to 48 h. Using LC-MS methods, serum samples from NHPs were analyzed for the presence of human IgG and IgA. At none of the time points, human IgG and IgA were detectable. This indicates that systemic bioavailability of inhaled Igs may be considered very low on this basis.

### Biodistribution of nebulized Igs

**Fig. 4:**
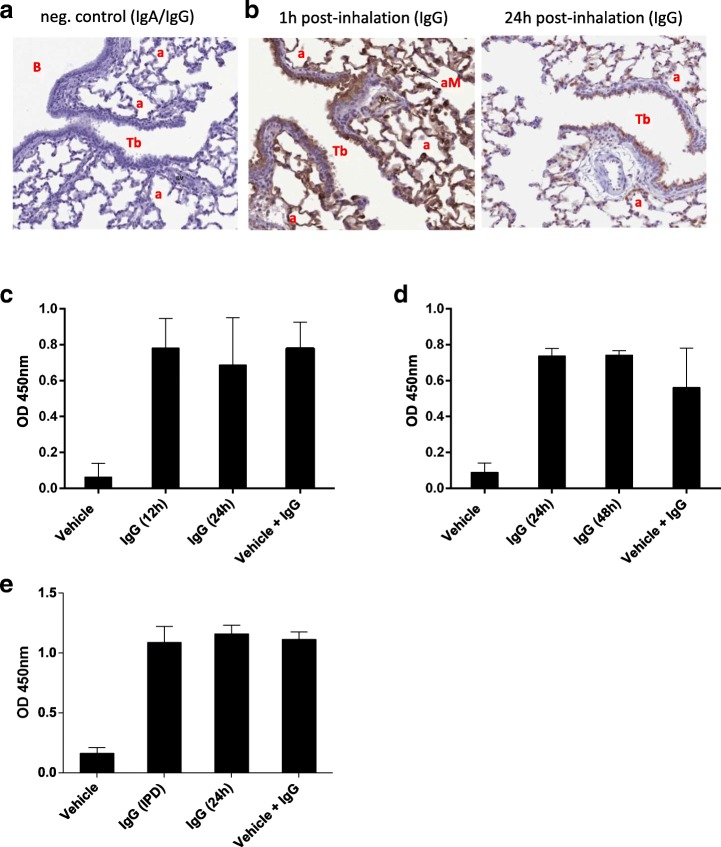
Topically applied nebulized IgG can reach the conducting airways and the alveoli in rats and remain functional over time. Lung tissue from IgG- and vehicle-treated animals were fixed and embedded in paraffin. Time points represented are 1 h and 24 h post-inhalation. Tissue sections were analyzed using a pan-Ig secondary antibody coupled to HRP. **a** Section of lung tissue obtained from a vehicle-treated animal is shown. It represents the level of background of the detection antibody. **b** Staining with detection antibody of sections obtained from tissues of animal treated with nebulized IgG is shown, legend for the red letters appearing in the sections is as follows; a: alveoli, aM: alveolar macrophage, B: bronchi, Tb: terminal bronchi. **c-f** Assessment of binding activity of nebulized IgG and IgA recovered from broncho-alveolar lavages was performed using ELISA. Tetanus toxoid antigen (**c**, **d**) was used to evaluate IgG from broncho-alveolar lavages obtained from rats (**c**) and non-human primates (**d**). Pneumococci polysaccharides were also used to evaluate IgG (**e**) for broncho-alveolar lavages obtained from non-human primates

To identify the compartments of the lungs that were reached by nebulized Igs, immunohistochemistry was conducted on lung sections from treated rats. Staining of lung sections from vehicle-treated animals confirmed that detection antibodies were not cross-reacting with endogenous Igs (Fig. 4a). Human IgG was detected in the lining of the trachea (data not shown) and bronchi, as well as in the periphery of the alveoli within the lungs (Fig. 4b). In line with the BAL PK data, a stronger signal was observed at 1 h post-administration than at 24 h, with the same tissue location. Interestingly, there was no detectable signal in the lamina propria of the lung tissues at 1 h or 24 h post-inhalation. Similarly, human IgG and IgA were detected in NHP lung tissue sections collected 72 h after the last (4th) inhalation cycle (Additional file 1: Figure S4).

### Nebulized Igs remain active in situ

Mucosal environment can be deleterious for Igs due to the presence of proteases, e.g. IgA1-specific proteases [20, 24, 25]. Therefore, we tested the integrity and functionality of Ig samples recovered from rat BAL fluid 24 h, and from NHP BAL fluid 48 h after nebulization. No degradation of Igs was observed in either species (data not shown) indicating that Igs remained structurally sound in situ. To test antigen binding capacity of nebulized Igs we used ELISAs specific for tetanus toxoid or streptococcal polysaccharide as described above. IgG recovered from rat BAL fluid (Fig. 4c) or NHP BAL fluid (Fig. 4d and e) yielded an equivalent signal than the same amount of non-nebulized IgG spiked into fresh BAL fluid. These data indicate that Igs are still able to bind antigens 24-48 h after being deposited in the lungs.

### Delivered dose estimations in humans

Finally, for each Ig formulation, breath simulation experiments were conducted to estimate the potential delivered dose of a 2 ml IgG formulation nebulized from the device. Delivered dose was measured by collecting the nebulized product on an inhalation filter, which was placed between the nebulizer mouth piece and the pump. Respirable dose (RD) was further calculated by applying the frequency of droplets with a size below 5 μm to the delivered dose values. As expected from the aerosol characteristics reported in Table 1, delivered doses obtained for the 5% formulations were comparable, with values ranging from 49.36 to 50.92 mg (Table 4). With a 2-fold higher concentration, the delivered dose of the 10%-formulated IgG was almost double that of the 5%-formulated IgG. However, when the delivered dose was expressed as a percentage of the total amount used in the assay, 10 and 5% formulations gave close results (45.4 to 51.4%). Respirable dose was also very similar for all formulations. These data suggest that around 35% of the Igs (RD < 5 μm) could potentially deposit into human lungs (Table 4). The residual amounts of Igs in the device were also similar between all formulations and support the published values obtained with active vibrating membrane nebulizers [13].

**Table 4:** Estimated delivered and respirable doses upon nebulization of 200 mg of IgG. Breath simulation experiments and laser diffraction measurements were conducted upon nebulization of 2 ml (200 mg) of IgG to measure delivered and respirable doses (RD)

Formulation	Nebulized dose (mg)	Residue (%)	Delivered dose (mg)	Delivered dose (%)	RD < 5 μm (mg)	RD < 5 μm (%)
IgG 10%	200	32.7 ± 8.10	94.06 ± 18.14	45.4 ± 9.00	72.24 ± 13.64	34.91 ± 6.78
IgG 5%	100	33 ± 9.10	49.36 ± 7.37	47.7 ± 7.10	34.36 ± 4.91	33.21 ± 4.74
IgA 5%	100	30.4 ± 6.90	48.41 ± 6.73	48.9± 6.90	35.33 ± 4.59	35.69 ± 4.63
IgAM 5%	100	30.8 ± 6.00	50.92 ± 6.32	51.4 ± 6.30	38.96 ± 5.37	39.34 ± 5.29

### Prevention of acute respiratory tract infection

**Fig. 5:**
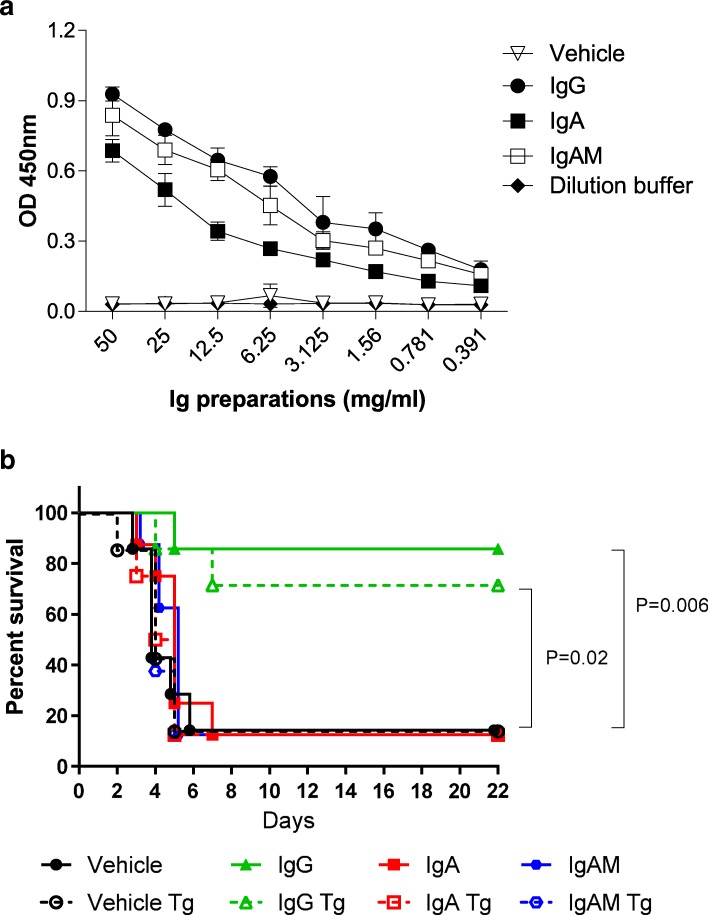
Topically applied human plasma-derived IgG protect mice against death in an acute *S. pneumoniae* infection model. Binding of IgG, IgA and IgAM preparations to *S. pneumoniae* A66.1 bacteria was assessed by ELISA (**a**). Survival of C57BL/6 WT and human CD89Tg mice infected with 5 × 10^5^ A66.1 *S. pneumoniae* at Day 0 (**b**). Vehicle or Ig preparations were given a day prior infection. *N* = 8 mice/group

To demonstrate that in principle topically applied immunoglobulins can prevent lung infection and to compare IgG and IgA-containing preparations, we used both C57BL/6 wild type (WT) and human CD89Tg mice in a *S. pneumoniae* lung infection model. Binding assay showed that all Ig preparations could recognize this strain. However, both IgG and IgAM preparations showed similar and higher titers against the bacteria than the IgA preparation (Fig. 5a).

Prophylactic intra-nasal application of human plasma-derived IgG 1 day prior to lethal infection led to 70–90% survival (Fig. 5b). In contrast, none of the IgA-containing preparations were able to protect mice from lethal infection, no difference between CD89-expressing and wild-type mice was observed (Fig. 5b).

## Discussion

Drug delivery directly into the respiratory tract to prevent or treat pulmonary diseases offers many benefits [26], such as the localized delivery of high drug concentrations to the affected [27–30]. Current therapies for COPD or PID patients do not completely protect against respiratory tract infections. To address this, we investigated the feasibility of the topical delivery of plasma-derived Igs directly into the lungs of patients via nebulization. Polyclonal, plasma-derived Igs manufactured from plasma pools of more than 1000 donors contain antibodies against a wide variety of bacterial and viral antigens. IgG-replacement therapy, for example, has been shown to be efficient at decreasing severe bacterial infections in PID patients, thus supporting the rationale for developing an inhaled version of the product [31].

The majority of plasma-derived Ig products on the market contain highly purified IgG. Other Ig isotypes that are naturally present in the respiratory tract, such as monomeric IgA and polymeric Igs (i.e. dimeric IgA and polymeric IgM) [9, 32] are usually excluded from these products but can be obtained by plasma sub-fractionation. While their relative efficacies at preventing respiratory tract infections remain to be addressed, our data provide important insights into the feasibility of nebulizing several Ig isotypes, including polymeric forms, and into the extent of their deposition into mammalian lungs.

Plasma-derived Igs are prepared in highly concentrated formulations (5–20%) for intravenous and subcutaneous delivery. The successful nebulization of low concentration IgG (up to 4%) has been reported [33] but, to our knowledge, we are the first to describe the robust and reproducible generation of aerosols from polyclonal Ig formulations at concentrations of 10% IgG. Moreover, the total output rate obtained for each formulation was high, which allowed for the rapid nebulization of Igs (2 ml within 3.5 min). Importantly, polymeric Igs (IgAM) at 5% final concentration could also be nebulized, despite the higher viscosity (3.74 mPa.s) of these preparations than a 10% IgG solution (3.20 mPa.s). In particular, the structure of a huge protein such as IgM was not affected. At last, nebulization is dependent on viscosity, and other devices have been unable to nebulize solutions with a viscosity higher than 2.74 mPa.s [34].

Optimal lung deposition is obtained for droplets with a diameter comprised between 5 and 1 μm [14, 35]. We showed that aerosol clouds generated from 4 different Ig formulations displayed similar, if not identical characteristics. Importantly, MMD obtained for each formulation was below 4 μm but above 1 μm, with a respirable fraction above 69%. Using 2 ml of each formulation (100–200 mg Ig) in breathing experiments, we extrapolated that the respirable dose would be approximately 35% in humans, which corroborate published data [22, 36]. While this number may seem low compared to the 100% dose available in systemic circulation after intravenous injection, it reflects a very good outcome with current state-of-the-art nebulizing technology. For instance, with older jet nebulizers only ~ 12% of the delivered dose reach the lung and best nebulizer achieve up to 41% delivered dose [36, 37]. Several factors explain this outcome; presence of dead volume in the reservoir which account for a certain loss of the product, limitation to droplets below 5 μm to enter the lungs (bigger droplets will impact the mouth or throat), and exhalation of some of the aerosol droplets during breathing.

Animal studies were performed to explore the feasibility of topically applying plasma-derived Igs using nebulizers into the upper and lower airways of normally breathing, conscious animals, without invasive tubing. Both rat and NHP studies successfully demonstrated the deposition of human IgG- and IgA-containing preparations into the conducting airways as well as the alveoli of treated lungs. Interestingly, the amount of human Igs found in the BAL of NHP were higher, which certainly points out the differences in breathing physiology and the size of the airways between the two species. Rodents are obligate nose-breathers while NHP can breathe through both the nose and mouth.

The detection of human Ig in the BAL of NHP 48 h post-inhalation is a significant observation as inhaled particles are normally cleared rapidly from the lungs. Mucociliary clearance and alveolar macrophages are primarily involved in this process [25]. Mucociliary clearance functions by moving the mucus present in the bronchi out of the respiratory tree and alveolar macrophages act as a primary barrier to macromolecule absorption in the lungs [38]. Human Igs were detected in the BAL from rats and NHP at 24 h and 48 h post-inhalation, respectively, as well as on sections 72 h post-dose, and indicate the successful deposition of large amounts of Igs into the lungs of these animals. Nevertheless, weekly inhalations did not lead to an accumulation of nebulized Igs when measured immediately post-dose. This suggests that the amount of nebulized Igs remaining in the lungs 7 days post-inhalation was negligible, in line with published results obtained in mice with an inhaled anti-VEGF antibody [39].

As mucus turnover is considered to take place over 24 h, it is not clear whether the nebulized Igs detected in the BAL of rats and NHP at 24 h and 48 h post-dose, respectively, account for the Igs remaining in the alveolar space or the bronchi. IHC data from the rat study suggest that there are still detectable amounts of human IgG in the terminal bronchi 24 h post-deposition. This may be a result of the mucociliary clearance in rats not being able to remove all the deposited human Ig present on the bronchi within 24 h, or it may reflect the presence of xenoreactive antibodies in the formulations, which would remain bound to the epithelium. Future studies should assess the clearance rate of inhaled Ig from the human bronchi.

The amount of nebulized Ig that was absorbed through the lung epithelium remains uncertain. From the IHC data, IgG could not be detected in the epithelium of the lungs at 24 h post-dose. Bioavailability of nebulized Igs in the systemic circulation was also addressed. No human IgG1 nor IgA1 could be detected in all serum samples collected from NHPs (LLOQ values for IgG1 and IgA1 being of 5.64 μg/ml and 16.09 μg/ml respectively). Nevertheless, the absence of detectable Ig levels suggests that, if at all, only small amounts of Ig were systemically absorbed after nebulization.

The hydrophilic and lipophilic properties of proteins render them susceptible to adsorption or aggregation at interfaces. In this present study, we did not detect any increase in aggregates after nebulization despite the use of Ig preparations that were highly concentrated. Nevertheless, potential immune-complex mediated adverse effects will need to be carefully monitored in initial human studies, especially in patients with underlying lung damage and lung inflammation.

At mucosal surfaces, Igs prevent pathogen adhesion and invasion through immune exclusion [32, 40]. Using antigens from respiratory tract pathogens or a specific *Shigella flexneri* infection model, we showed that antigen binding was unaffected after nebulization, despite nebulizing highly concentrated Igs of monomeric or polymeric forms, and that nebulized polymeric Igs retained their ability to bind and agglutinate bacteria. Finally, the Fc portions of nebulized Ig preparations were also found to be fully functional at triggering respiratory burst in human neutrophils in complexed form. Overall, these results demonstrate that nebulization of monomeric and polymeric Igs at high concentrations with the eFlow® nebulizer had no impact on their structure or function.

To obtain further support for administering nebulized plasma-derived Igs into patients, we assessed the ex vivo activity of nebulized Igs recovered from the BAL of NHP and rats using ELISAs measuring the binding of human IgG to tetanus toxoid antigen and pneumococci polysaccharides, to lower endogenous Ig (e.g. in vehicle-treated animals) background signal. We could verify that nebulized Igs appeared fully functional in the lungs of the animals, even when their activity was assessed 48 h post-deposition, suggesting a low catabolic activity in the lungs. Altogether, we showed that highly concentrated Igs retain their functions upon nebulization and in situ for at least 48 h.

Respiratory tract infections affect millions of patients each year, with at-risk groups including COPD, NCFB and PID patients. To prevent these infections in PID patients, prophylactic use of antibiotics is often prescribed in combination with IgG replacement therapy [41]. While antibiotics have proven to be efficacious at reducing bacterial infections, the spread of antibiotic-resistant bacterial strains is a concern particularly in PID patients [42]. Passive immunization with topically applied plasma-derived Igs may be an attractive alternative to antibiotics to prevent respiratory tract infections from bacterial as well as viral origins.

In this study, we show that human plasma-derived immunoglobulin G given prophylactically display a high degree of protection against an acute *S. pneumoniae* respiratory infection in mice. *S. pneumoniae* is a human pathogen, which remains a leading cause of serious illness among infants, immunocompromised patients, and the elderly population [43]. It infects the host mainly through the respiratory tract and can cause life-threatening diseases, such as pneumonia, bacteremia, and meningitis when reaching the circulation [43, 44]. The large number of pneumococcal serogroups is a hurdle for the design of effective vaccines [45]. Alternative approaches such as the use of plasma-derived immunoglobulins may represent an optimal protection strategy against this pathogen. The model of *S. pneumoniae* infection in mice was chosen for its close mimic to the human disease. Innate immunity provides protection against low doses of bacteria (up to 10^5^ in mice). However, higher doses escape immunity with the bacteria becoming invasive and leading to fatal sepsis. In support of this fact, 90% of vehicle treated animals died 6 days after receiving the bacteria (Fig. 5b).

Topical application of nebulized drugs into the airways of mice is challenging as rodents have complex turbinates, which efficiently filter aerosols. Moreover, accurate dosing of mice in nebulization chambers is not feasible as aerosols will also deposit on their fur and can be potentially licked. To investigate the efficacy of human-plasma derived immunoglobulins in our infection model, we therefore directly deposited the Ig preparations into the nares of anesthetized mice using a pipette.

IgA-containing preparations did not show efficacy in this model. IgA showed slightly lower titers against the A66.1 bacteria than IgG, which could explain this outcome. However, IgAM had similar titers as IgG. Severity of the infection model or the type of pathogen used may account for this difference in efficacy, particularly since IgG-mediated opsonophagocytosis has been described as the optimal correlate of protection against pneumococcal infection [46]. Secretion of IgA1 proteases by *S. pneumoniae* strains could also potentially explain the lack of protection by human-plasma-derived IgA, which are mainly IgA1 [47]. Further evaluation of the efficacy of these immunoglobulins to protect against respiratory pathogens is needed.

## Conclusions

We are demonstrating the feasibility to nebulized highly concentrated plasma-derived immunoglobulins into animal lungs without loss of their functions. Nebulization of a 10% (100 mg/ml) protein solution such as IgG has, to our knowledge, never been shown before. In addition, we showed that nebulization process did not harm multimeric immunoglobulins (dimeric IgA and pentameric IgM), an information which was unknown until now. At last, and importantly, our study did not use any artificial delivery system or procedure (e.g. microsprayer or intra-tracheal delivery) to increase deposition in animal. Data on deposition and distribution of inhaled plasma-derived immunoglobulins in animal lungs reflects real breathing conditions and therefore real deposition. We showed that nebulized Immunoglobulins could be distributed in both rat and NHP lungs from the bronchi to the alveoli. Topically applied immunoglobulins also protected mice against an acute bacterial respiratory infection. This indicated that the approach to use nebulized immunoglobulins topically into the lungs to prevent infections in human is feasible. Formal non-clinical safety studies to determine the maximal tolerable dose followed by cautions first-in-human safety studies will be the logical next development steps.

## Additional file

Additional file 1: **Figure S1.** Aerosol characterization upon nebulization of distinct Ig formulations. PBS and proline formulations of diverse Ig preparations were nebulized side by side. Mass median diameter (A), total output rate (B) and respirable fraction (C) were measured using laser diffraction. **Figure S2.** Chromatograms of IgA and IgAM separated by size exclusion. A.B. Chromatograms of IgA (5%) (A) and IgAM (5%) (B) separated by size exclusion are represented. Blue chromatograms refer to the nebulized (N) formulation. Red chromatograms refer to the non-nebulized, or control (Co), formulation. **Figure S3.** Nebulized Igs retain their function in an in vitro model of Shigella infection. Polarized Caco-2 cell monolayers were infected overnight with Shigella flexneri alone or in presence of nebulized or non-nebulized Ig formulations. A. Quantitative analysis of infected areas was determined from laser scanning confocal microscopy pictures using ImageJ software. B-D. Basolateral secretion of TNF-α (B), CXCL8 (C), and CCL3 (D) was measured by ELISA. Non-infected Caco-2 cell monolayers served as a control. **Figure S4.** Lung tissue from IgG, IgA, IgAM- and vehicle-treated animals (NHP) were collected 72 h post-exposure (4th inhalation) and were fixed and embedded in paraffin. Tissue sections were analyzed for IgG (A) and IgA (B) using specific secondary antibody coupled to HRP. For each detection antibody, detection of lung tissue obtained from a vehicle-treated animal is shown. It represents the level of background of the detection antibody. (PDF 717 kb)

## Abbreviations

BAL: Broncho-alveolar lavage
COPD: Chronic obstructive pulmonary disease
DLS: Dynamic light scattering
DPI: Dry powder inhaler
ELISA: Enzyme-linked immunosorbent assay
Fab: Antigen-binding fragment
Fc: Fragment crystallizable region
GSD: Geometric standard deviation
HMS: High molecular species
HSC: Hematopoietic stem cell
Ig(s): Immunoglobulin(s)
IVIG: Intra-venous immunoglobulin G
LC-MS: Liquid chromatography–mass spectrometry
MMD: Mass median diameter
NCFB: Non-CF bronchiectasis
NHP: Non-human primates
pd.: Plasma-derived
PID: Primary immunodeficiency diseases
pIgR: Polymeric immunoglobulin receptor
pMDI: Pressurized metered dose inhaler
PS80: Polysorbate 80
RF: Respirable fraction
*S. pneumoniae*: *Streptococcus pneumoniae*
SE-HPLC: Size exclusion high-performance liquid chromatography
sFcγR: Soluble Fc receptor for immunoglobulin G
sIgA: Secretory IgA
TOR: Total output rate
TTA: Tetanus toxoid antigen
