# Supplementary figures and images for: Topical application of nebulized human IgG, IgA and IgAM in the lungs of rats and non-human primates
Source: Respir Res. 2019 May 22;20:99. doi: 10.1186/s12931-019-1057-3 (PMC6532128; doi:10.1186/s12931-019-1057-3)

# Additional Figure 1

A

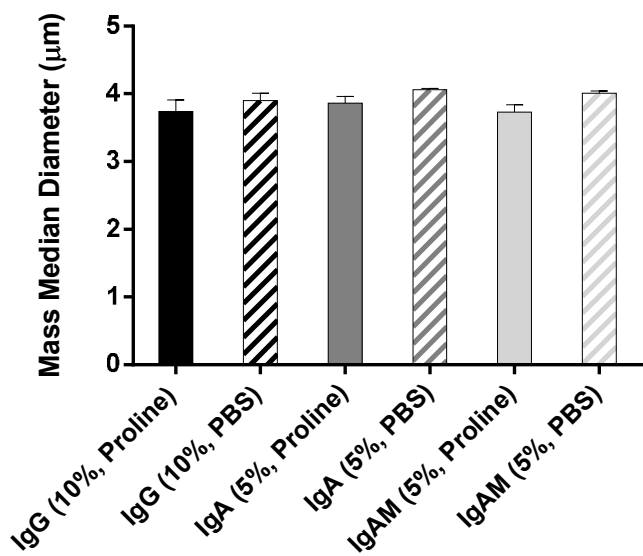

B

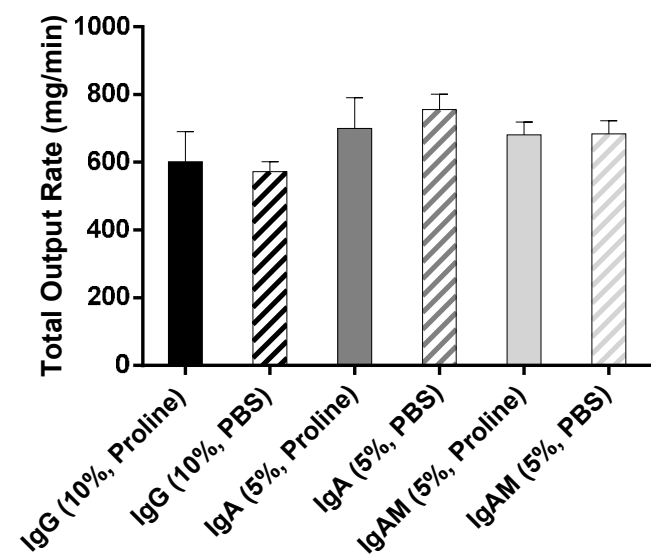

C

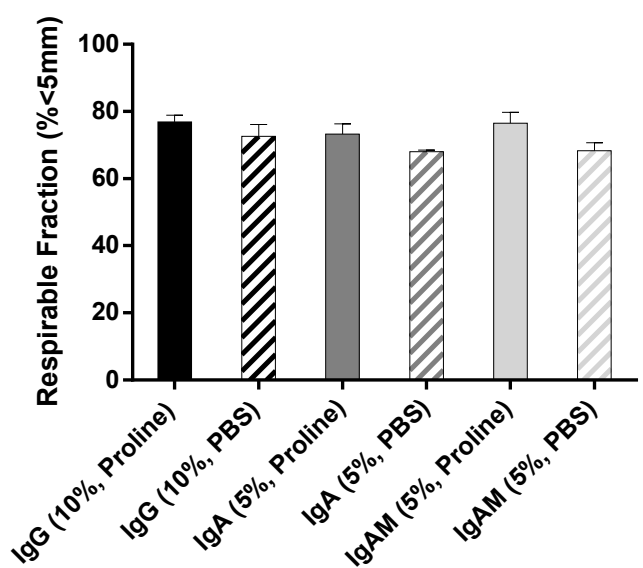

# Additional Figure 2

A

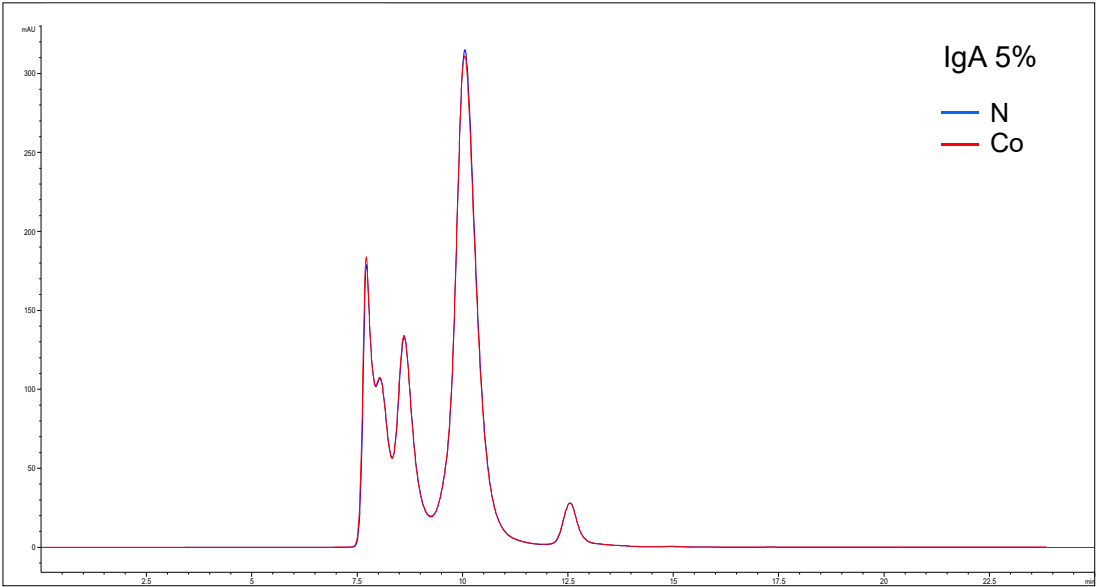

B

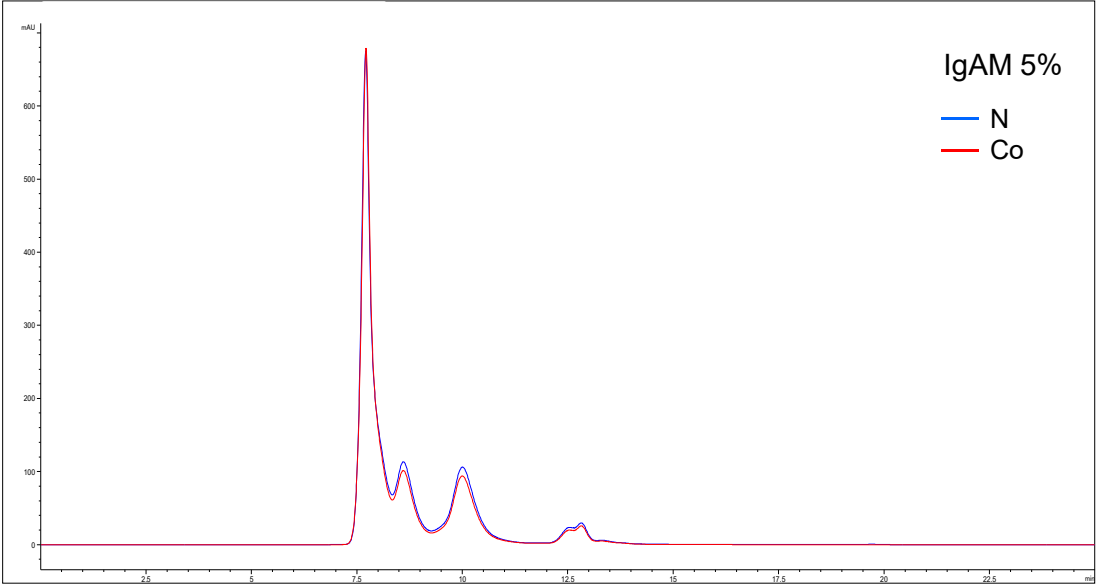

# Additional Figure 3

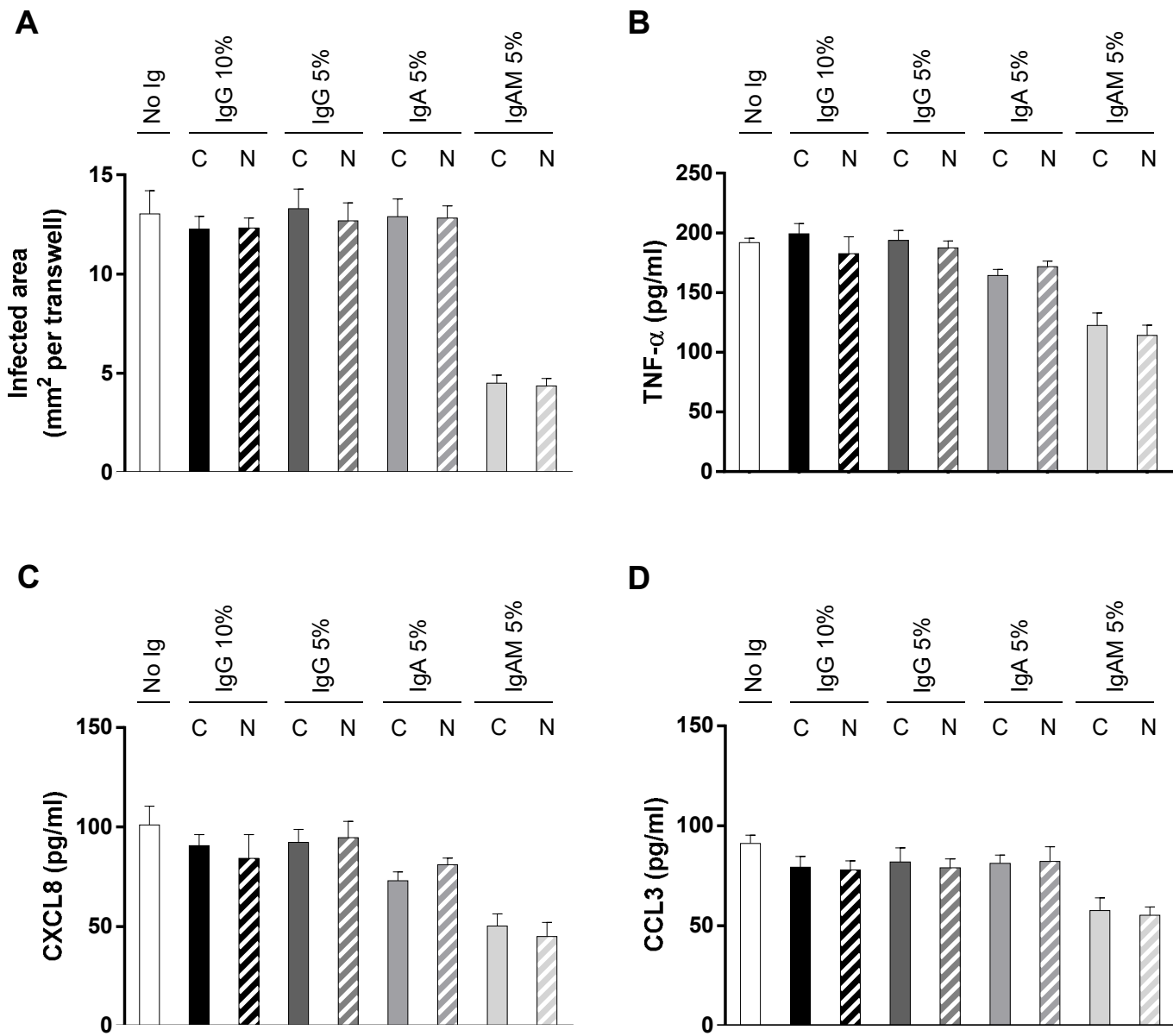

# Additional Figure 4

A

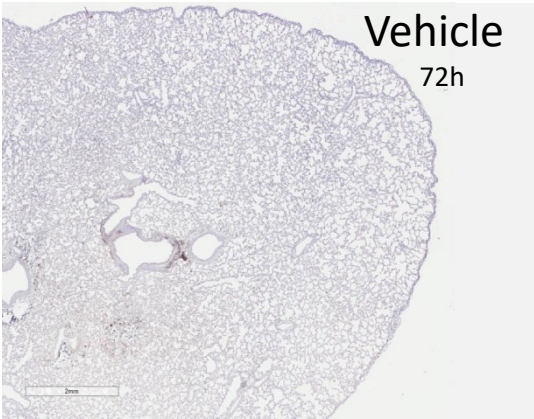

B

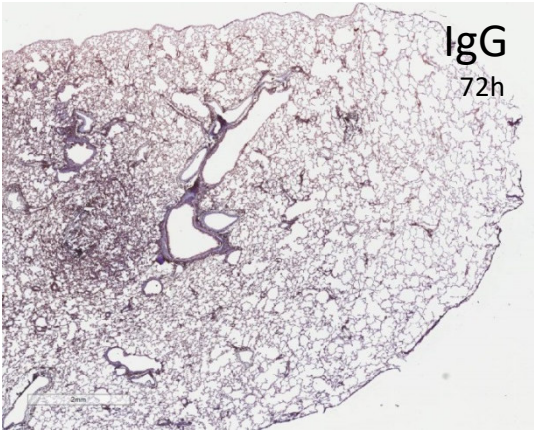

C

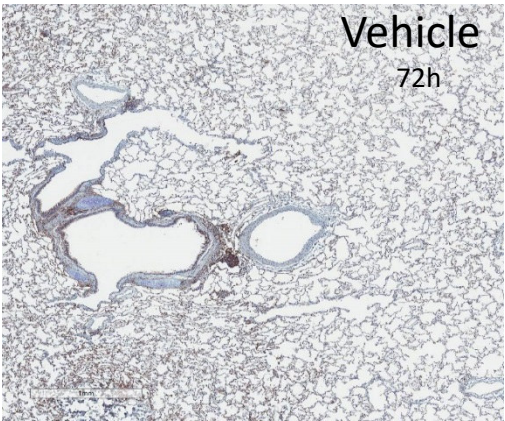

D

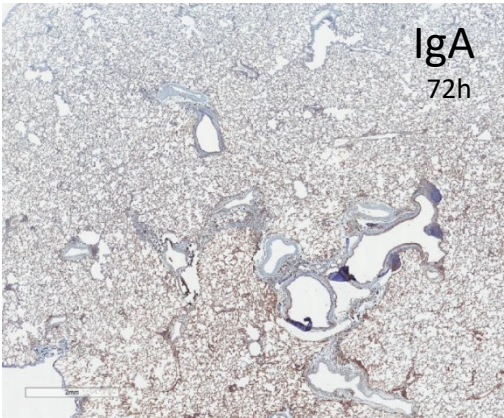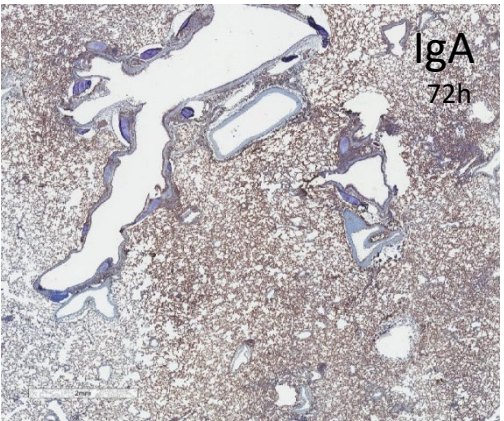

Supplement: Supplementary file 1 — Figure S1. Aerosol characterization upon nebulization of distinct Ig formulations. PBS and proline formulations of diverse Ig preparations were nebulized side by side. Mass median diameter (A), total output rate (B) and respirable fraction (C) were measured using laser diffraction. Figure S2. Chromatograms of IgA and IgAM separated by size exclusion. A.B. Chromatograms of IgA (5%) (A) and IgAM (5%) (B) separated by size exclusion are represented. Blue chromatograms refer to the nebulized (N) formulation. Red chromatograms refer to the non-nebulized, or control (Co), formulation. Figure S3. Nebulized Igs retain their function in an in vitro model of Shigella infection. Polarized Caco-2 cell monolayers were infected overnight with Shigella flexneri alone or in presence of nebulized or non-nebulized Ig formulations. A. Quantitative analysis of infected areas was determined from laser scanning confocal microscopy pictures using ImageJ software. B-D. Basolateral secretion of TNF-α (B), CXCL8 (C), and CCL3 (D) was measured by ELISA. Non-infected Caco-2 cell monolayers served as a control. Figure S4. Lung tissue from IgG, IgA, IgAM- and vehicle-treated animals (NHP) were collected 72 h post-exposure (4th inhalation) and were fixed and embedded in paraffin. Tissue sections were analyzed for IgG (A) and IgA (B) using specific secondary antibody coupled to HRP. For each detection antibody, detection of lung tissue obtained from a vehicle-treated animal is shown. It represents the level of background of the detection antibody. (PDF 717 kb) [file 12931_2019_1057_MOESM1_ESM.pdf]
